# Zoonotic *Onchocerca lupi* Infection in Dogs, Greece and Portugal, 2011–2012

**DOI:** 10.3201/eid1912.130264

**Published:** 2013-12

**Authors:** Domenico Otranto, Filipe Dantas-Torres, Alessio Giannelli, Maria Stefania Latrofa, Elias Papadopoulos, Luís Cardoso, Helder Cortes

**Affiliations:** Università degli Studi di Bari, Valenzano, Italy (D. Otranto, F. Dantas-Torres, A. Giannelli, M.S. Latrofa);; Aggeu Magalhães Research Institute, Recife, Brazil (F. Dantas-Torres); Faculty of Veterinary Medicine, Thessaloniki, Greece (E. Papadopoulos);; University of Trás-os-Montes e Alto Douro, Vila Real, Portugal (L. Cardoso);; Institute for Molecular and Cell Biology, Oporto, Portugal (L. Cardoso);; University of Évora, Evora, Portugal (H. Cortes)

**Keywords:** Onchocerca lupi, dogs, zoonoses, infection, Portugal, Greece, parasites

## Abstract

*Onchocerca lupi* infection is reported primarily in symptomatic dogs. We aimed to determine the infection in dogs from areas of Greece and Portugal with reported cases. Of 107 dogs, 9 (8%) were skin snip–positive for the parasite. DNA sequences of parasites in specimens from distinct dog populations differed genetically from thoses in GenBank.

Zoonotic onchocercosis has been attributed to species that primarily infest cattle (*Onchocerca gutturosa*), horses (*O. cervicalis*), the European deer (*O. jakutensis*), and wild boars (*O. dewittei japonica*) (*1*). In their definitive hosts, all these species localize in subcutaneous tissues, muscular fasciae, or cervical ligaments, whereas in humans, *O. gutturosa* and *O. cervicalis* also have an ocular localization (reviewed in *2*).

*O. lupi* is a recently recognized parasite causing nodular lesions associated with ocular disease (i.e., conjunctivitis, ocular swelling, photophobia, lacrimation, discharge, exophthalmia) in dogs (*3*). The zoonotic potential of this filarioid has been suspected (*4*) but has only recently been demonstrated in a patient from Turkey (*5*). Ocular cases in humans are increasingly being reported worldwide, including in Iran (*6*), Turkey, and Tunisia (*7*). In addition, *O. lupi* infection was recently diagnosed near the spinal canal in a 22-month-old child from Arizona, USA (*2*).

Since its first description in a Caucasian wolf (*Canis lupus*) from Georgia in 1967 (*8*), *O. lupi* remained almost unknown for decades until being reported in dogs from southern Europe (Greece, Portugal) and central Europe (Germany, Hungary) (Figure 1, panel A) (reviewed in *3*). In the western United States, canine onchocercosis (*9*) has been attributed to species parasitizing other hosts (i.e., cattle, horses, or wild ungulates), but such cases were probably caused by *O. lupi*, as recently confirmed morphologically and molecularly in 2 cats (*10*) and 4 dogs (*11*).

**Figure 1:**
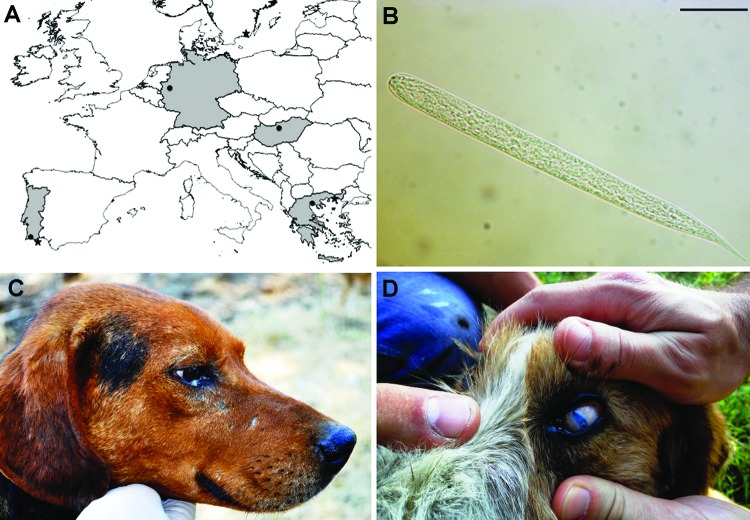
A) Areas (in gray) and localities (black dots) where *Onchocerca lupi* infections were reported and sampling sites (asterisk) from Greece (site A) and Portugal (site B). Scale bar = 500 km. B) Light microscopy image of microfilariae of *O. lupi* detected at the skin sediment. Scale bar = 20 µm. Original magnification ×100. Dog positive for *O. lupi* had conjunctival swelling and a purulent mucus discharge (C), or blindness with severe keratitis and uveitis (D).

Several aspects of the biology and ecology of *O. lupi* remain unknown and the knowledge of its actual distribution is limited to a few case reports. We conducted an epidemiologic survey to estimate the occurrence of *O. lupi* infection in dog populations from areas of Greece and Portugal where multiple (*12*) or single (*13*) cases, respectively, have been reported. The genetic make-up of the parasites in the specimens collected from both canine populations was assessed by comparing them with sequences available in GenBank.

## The Study

In June 2011 and November 2012, we sampled a total of 107 dogs of different ages, sexes, and weights from a site in Greece (site A, 23 dogs) and a site in Portugal (site B, 84 dogs). Briefly, site A was located within the boundaries of Amaxades (25°04’27”E, 41°07’12”N, altitude 56 m), a small village of ≈1,000 inhabitants between Xanthi and Komotini, on the border between Greece and Turkey. This is a traditionally agricultural and poor dry area, with tobacco and sunflower plantations among the most important cultivations. Site B was in Olhão (southern Portugal, 7°50’33”O, 37°01’42” N, altitude 8 m), a municipality with 45,000 inhabitants representing ≈10% of the population of the Algarve, a region in which tourism is a major economic activity (Figure 1, panel A). Both sites are located along or near the seacoast, where small river streams run during the rainy season and dry out during the summer. Animals from site A were shepherd dogs living in small rural communities with sheep and goats; animals from site B were stray dogs kept according to Portuguese regulations in a shelter until they were adopted or euthanized. The shelter was surrounded by a large area of salt water in open facilities at which sea salt was collected.

Skin samples were collected by using a disposable scalpel over an area of ≈0.2 × 0.2 × 0.2 cm from inter ocular frontal area of the head and soaked at 37°C in saline solution for 1 h. Sediments (20 μL) were individually observed under light microscopy (i.e., 1 field of 18 × 18 mm coverslip). Microfilariae were counted, identified according to morphologic keys (*3**,**14*), and differentiated from those of filarioid species most commonly retrieved in dogs from the Mediterranean region (*15*). Briefly, microfilariae of *O. lupi* had a short flattened unsheathed body (mean length 110.1 ± 7.5 μm, width 6.8 ± 1.2 μm) with a rounded head bearing a tiny tooth on the cephalic edge. The body was blunt with a short bent tail of ≈11.7 μm (Figure 1, panel B).

Of 107 dogs, 9 (8%; 2 from site A and 7 from site B) were positive for *O. lupi* microfilariae, with a maximum of 480 microfilariae detected in a single sample. Animals positive for *O. lupi* at site A displayed a range of ocular alterations from conjunctival swelling and mucopurulent discharge (Figure 1, panel C) to blindness (Figure 1, panel D). Conversely, all animals from site B were asymptomatic.

**Figure 2:**
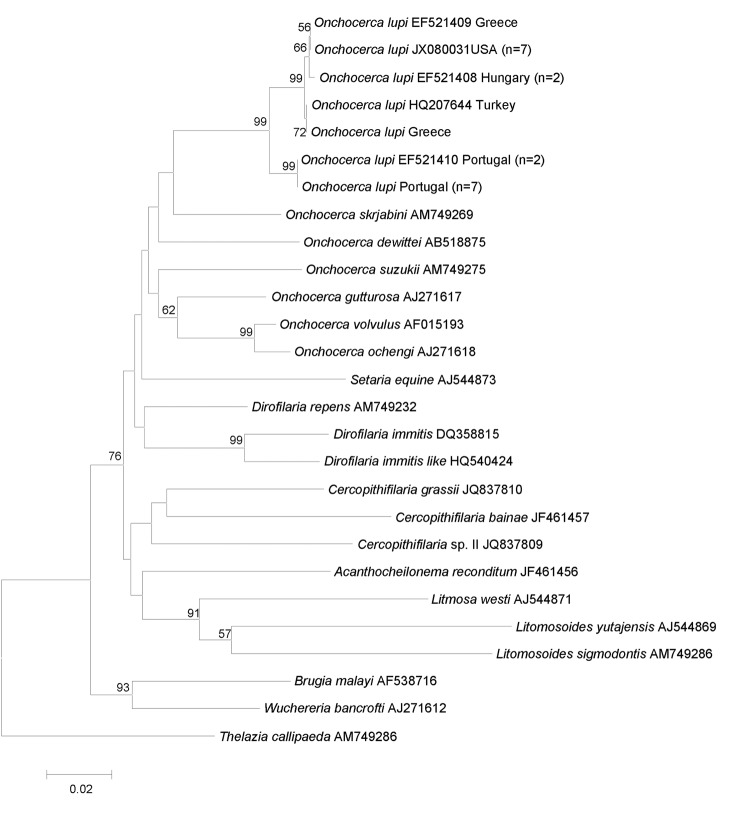
Phylogeny of *Onchocerca lupi* and other filarial nematodes based on cytochrome *c* oxidase subunit 1 gene sequences. *Thelazia callipaeda* was used as outgroup. Bootstrap confidence limits (8,000 replicates). GenBank accession numbers and number of haplotype sequences (in parenthesis) are reported along with their geographic origin. Scale bar indicates genetic differences.

After microscopic observations, microfilariae were removed with a 10-μL pipette and placed in saline solution in single tubes at –20°C, before DNA was extracted and partial (582 bp) cytochrome *c* oxidase subunit 1 (*cox*1) and 12S rDNA (304 bp) gene fragments amplified as described elsewhere (*7*). In accordance with the morphologic identification, the BLAST analysis (http://blast.ncbi.nlm.nih.gov/Blast.cgi) of both genes showed a high overall nucleotide homology with sequences of *O. lupi* available in GenBank (i.e., 99% for 12S rDNA: GU365879; from 98% to 100% for *cox*1 accession numbers reported in Figure 2).

All *cox*1 sequences available in GenBank for *O. lupi* were analyzed by using MEGA5 (www.megasoftware.net) and showed a low intraspecific variability (mean 0.7%, range 0%–2.1%). All *cox*1 sequences of *O. lupi* were identical according their geographic provenience (i.e., 2 in Greece, 1 in Turkey, 2 in Hungary, 9 in Portugal, and 7 in the United States) and had a high nucleotide similarity (mean 99.2%, range 99.6%–100%), except for those from Portugal, which differed considerably from the others (mean 98.2%, range 97.9%–98.2%). The phylogenetic analysis using *cox*1 sequences by MEGA5 under the neighbor-joining method confirmed that *O. lupi* clustered with those in the genus, to the exclusion of other filarioids. In particular, specimens of *O. lupi* from Portugal formed a sister clade (with a strong nodal support) with those from other origins (Figure 2). Sequences were deposited in GenBank under accession numbers KC686701–KC686702 and KC686703–KC686704 for *cox*1 and 12S rDNA, respectively).

## Conclusions

Our data clearly showed that *O. lupi* infection occurred in dogs from both sampling areas (overall positivity 8%) and that dogs exhibited different clinical features that ranged from no apparent clinical sign to blindness. The lack of any ocular lesions in *O. lupi*–infected dogs from site B might be due to differences in sampling times (i.e., during summer, site A, and late autumn, site B), and thus in adult worm development and/or in the pathogenicity of the populations of parasites, which might reflect the variations documented in *cox*1 sequences of individuals from Portugal and those populations of *O. lupi* from different geographic areas. In addition, aberrant infection of adult nematodes in dogs cannot be ruled out, as recently demonstrated in a human patient from Arizona in whom *O. lupi* was found in the spinal canal (*2*). Whether asymptomatic animals can be a source of *O. lupi* infection for the vectors (which remain unknown) needs to be assessed (*16 *in Technical Appendix). Despite the increasing number of *O. lupi* infections reported in animals and humans, the difficulties in achieving a reliable diagnosis through the skin-snip technique and the unwillingness of some pet owners to allow collection of a piece of skin from the animal’s head, might explain the scant data on *O. lupi*. Therefore, population-based surveys should be performed to estimate the distribution of the infection in dogs and to assess the risk to humans. In addition, further studies are needed to improve understanding of the biology of this parasite, including its hosts and vectors. The reliability of the tools and procedures for diagnosing *O. lupi* infection in dogs and in humans, especially in asymptomatic individuals, also needs to be assessed. Finally, our data should alert physicians and ophthalmologists about the potential risk for *O. lupi* infection in humans and their pets (cats and dogs).

Technical Appendix Additional reference.

## References

[1] Otranto D, Eberhard ML. Zoonotic helminths affecting the human eye. Parasit Vectors. 2011;4:41. 10.1186/1756-3305-4-41 PMC3071329 21429191

[2] Eberhard ML, Ostovar GA, Chundu K, Hobohm D, Feiz-Erfan I, Mathison BA, Zoonotic *Onchocerca lupi* infection in a 22-month-old child in Arizona: first report in the United States and a review of the literature. Am J Trop Med Hyg. 2013;88:601–5 . 10.4269/ajtmh.12-0733 23382171 PMC3592550

[3] Sréter T, Széll Z. Onchocercosis: a newly recognized disease in dogs. Vet Parasitol. 2008;151:1–13 . 10.1016/j.vetpar.2007.09.008 17951007

[4] Sréter T, Széll Z, Egyed Z, Varga I. Subconjunctival zoonotic onchocerciasis in man: aberrant infection with *Onchocerca lupi?* Ann Trop Med Parasitol. 2002;96:497–502 . 10.1179/000349802125001267 12194710

[5] Otranto D, Sakru N, Testini G, Gürlü VP, Yakar K, Lia RP, Case report: first evidence of human zoonotic infection by *Onchocerca lupi* (Spirurida, Onchocercidae). Am J Trop Med Hyg. 2011;84:55–8. 10.4269/ajtmh.2011.10-0465 21212202 PMC3005520

[6] Mowlavi G, Farzbod F, Kheirkhah A, Mobedi I, Bowman DD, Naddaf SR. Human ocular onchocerciasis caused by *Onchocerca lupi* (Spirurida, Onchocercidae) in Iran. J Helminthol. 2013;6:1–6. 10.1017/S0022149X13000060 23388686

[7] Otranto D, Dantas-Torres F, Cebeci Z, Yeniad B, Buyukbabani N, Boral OB, Human ocular filariasis: further evidence on the zoonotic role of *Onchocerca lupi*. Parasit Vectors. 2012;5:84. 10.1186/1756-3305-5-84 PMC3407723 22541132

[8] Rodonaja TE. A new species of nematode, *Onchocerca lupi* n. sp., from *Canis lupus cubanensis.* Bulletin of the Academic of Science of Georgian SSR. 1967;45:715–9.

[9] Zarfoss MK, Dubielzig RR, Eberhard ML, Schmidt KS. Canine ocular onchocerciasis in the United States: two new cases and a review of the literature. Vet Ophthalmol. 2005;8:51–7 . 10.1111/j.1463-5224.2005.00348.x 15644101

[10] Labelle AL, Daniels JB, Dix M, Labelle P. *Onchocerca lupi* causing ocular disease in two cats. Vet Ophthalmol. 2011;14:105–10. 10.1111/j.1463-5224.2011.00911.x 21923832

[11] Labelle AL, Maddox CW, Daniels JB, Lanka S, Eggett TE, Dubielzig RR, Canine ocular onchocercosis in the United States is associated with *Onchocerca lupi.* Vet Parasitol. 2013;193:297–301. 10.1016/j.vetpar.2012.12.002 23276598

[12] Komnenou A, Eberhard ML, Kaldrymidou E, Tsalie E, Dessiris A. Subconjunctival filariasis due to *Onchocerca* sp. in dogs: report of 23 cases in Greece. Vet Ophthalmol. 2002;5:119–26 . 10.1046/j.1463-5224.2002.00235.x 12071870

[13] Faísca P, Morales-Hojas R, Alves M, Gomes J, Botelho M, Melo M, A case of canine ocular onchocercosis in Portugal. Vet Ophthalmol. 2010;13:117–21. 10.1111/j.1463-5224.2010.00763.x 20447031

[14] Mutafchiev Y, Dantas-Torres F, Giannelli A, Abramo A, Papadopulos E, Cardoso L, Redescription of *Onchocerca lupi* (Spirurida: Onchocercidae), with histopathological observations. Parasit Vectors. 2013. In press. 10.1186/1756-3305-6-309 PMC3818983 24499611

[15] Otranto D, Brianti E, Dantas-Torres F, Weigl S, Latrofa MS, Gaglio G, Morphological and molecular data on the dermal microfilariae of a species of *Cercopithifilaria* from a dog in Sicily. Vet Parasitol. 2011;182:221–9 . 10.1016/j.vetpar.2011.05.043 21705146

