# Supplementary material for: Zoonotic Onchocerca lupi Infection in Dogs, Greece and Portugal, 2011–2012
Source: Emerg Infect Dis. 2013 Dec;19(12):2000–3. doi: 10.3201/eid1912.130264 (PMC3840859; doi:10.3201/eid1912.130264)
Supplement: Technical Appendix — Additional reference. [file 13-0264-Techapp-s1.pdf]

Article DOI: http://dx.doi.org/10.3201/eid1912.130264

Zoonotic *Onchocerca lupi* Infection in Dogs, Greece and Portugal, 2011–2012

Technical Appendix

Additional Reference

16. Otranto D, Dantas-Torres F, Papadopoulos E, Petrić D, Ćupina AI, Bain O. Tracking the vector of *Onchocerca lupi* in a rural area of Greece. Emerg Infect Dis. 2012;18:1196–1200. PubMed http://dx.doi.org/10.3201/eid1807.AD1807
